# Rational Design of Aptamer-Tagged tRNAs

**DOI:** 10.3390/ijms21207793

**Published:** 2020-10-21

**Authors:** Takahito Mukai

**Affiliations:** Department of Life Science, College of Science, Rikkyo University, 3-34-1 Nishi-Ikebukuro, Toshima-ku, Tokyo 171-8501, Japan; takahito.mukai@rikkyo.ac.jp

**Keywords:** genetic code, anticodon, pseudo-anticodon, amber suppression

## Abstract

Reprogramming of the genetic code system is limited by the difficulty in creating new tRNA structures. Here, I developed translationally active tRNA variants tagged with a small hairpin RNA aptamer, using *Escherichia coli* reporter assay systems. As the tRNA chassis for engineering, I employed amber suppressor variants of allo-tRNAs having the 9/3 composition of the 12-base pair amino-acid acceptor branch as well as a long variable arm (V-arm). Although their V-arm is a strong binding site for seryl-tRNA synthetase (SerRS), insertion of a bulge nucleotide in the V-arm stem region prevented allo-tRNA molecules from being charged by SerRS with serine. The SerRS-rejecting allo-tRNA chassis were engineered to have another amino-acid identity of either alanine, tyrosine, or histidine. The tip of the V-arms was replaced with diverse hairpin RNA aptamers, which were recognized by their cognate proteins expressed in *E. coli*. A high-affinity interaction led to the sequestration of allo-tRNA molecules, while a moderate-affinity aptamer moiety recruited histidyl-tRNA synthetase variants fused with the cognate protein domain. The new design principle for tRNA-aptamer fusions will enhance radical and dynamic manipulation of the genetic code.

## 1. Introduction

**Figure 1:**
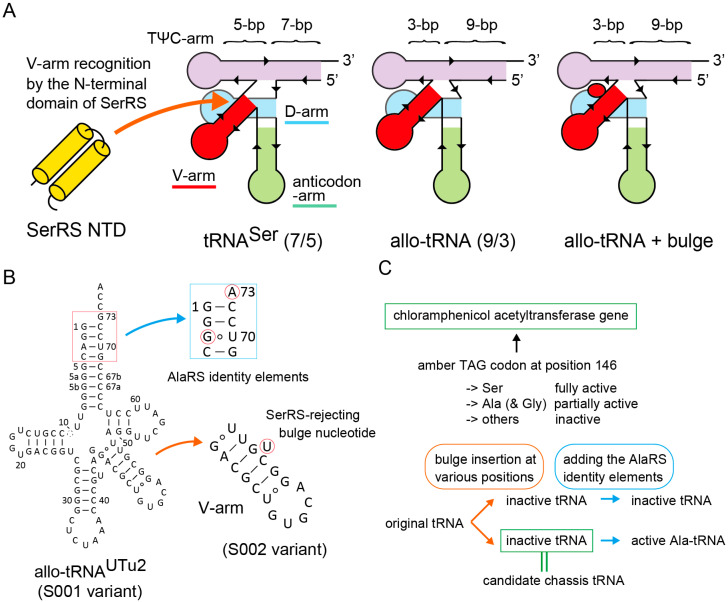
Strategy for tRNA chassis development. (**A**) Design of a new tRNA chassis derived from a few allo-tRNA species having the 9/3 composition. Compared to the *E. coli* tRNA^Ser^ having the canonical 7/5 composition, (9/3) allo-tRNA molecules may have a smaller space for the binding of the *N*-terminal domain (NTD) of SerRS. Thus, it was assumed that the interaction between SerRS and allo-tRNA molecules could be sterically hindered by the insertion of a bulge (flipped-out) nucleotide into the stem region of the V-arm. (**B**) A preliminary result suggested that the allo-tRNA^UTu2^ variant containing the bulge U was a poor substrate for SerRS, while its variant having the AlaRS identity elements was active for Ala-tRNA regardless of the bulge U insertion. (**C**) Experimental design for screening candidate chassis tRNAs by using a reporter gene in *E. coli*.

RNA aptamers are small RNA molecules that specifically bind to their target molecules. Small hairpin RNA aptamers are naturally found in certain mRNAs [1,2,3] and other RNAs [4]. Diverse hairpin aptamers have been used for tagging many kinds of RNA molecules for recruiting their cognate target proteins or ligands on the fusion RNA molecules [4,5,6,7]. On the other hand, transfer RNAs (tRNAs) are small RNA molecules that transfer proteinogenic amino acids onto the growing polypeptide chain in the ribosome. tRNAs fold into the L-shape tertiary structure, while some tRNAs have an extra arm in varying size (variable arm or V-arm) (see Figure 1). Although several types of tRNA variants fused with hairpin RNA aptamers have been developed [7,8,9,10,11,12,13], these fusion RNAs usually lost the translational activity of tRNA. Recently, “V-Spinach tRNAs” were developed by inserting a Baby Spinach aptamer [14] into the V-loop of a few *E. coli* tRNA species [8], although the V-Spinach tRNAs showed only marginal tRNA activities [8,15]. More recently, the V-arm hairpin of a yeast serine tRNA species was replaced by the MS2 phage hairpin RNA (19 nucleotides in length) to recruit an enzyme fused with the MS2 coat protein on the premature form of these fusion RNA molecules in yeast cells [11]. These works encouraged us to design a new generation of tRNA-aptamer fusions in which a small hairpin aptamer constitutes the tip of the V-arm.

tRNA plays a central role in reprogramming the genetic code system and establishing new genetic codes [16,17,18]. In nature, the genetic code may have been expanded to acquire selenocysteine (Sec) and pyrrolysine (Pyl) as the 21st and 22nd amino acid, respectively. Interestingly, canonical tRNA^Sec^ has the largest tRNA structure, while tRNA^Pyl^ has the most compact tRNA structure [16], which may help them escape from non-cognate aminoacyl-tRNA synthetases (aaRSs). The genetic code was expanded in diverse organisms using synthetic biologists by developing various sets of orthogonal tRNA and aaRS pairs that do not cross-react with endogenous tRNAs and aaRSs and other orthogonal tRNA-aaRS pairs [17,18,19,20,21,22]. It has been a big challenge to artificially create orthogonal tRNA species with both high tRNA activity and high orthogonality in part due to saturation of recognition elements of tRNA [23]. One promising approach is “tRNA Extension (tREX)” [17] by which the tRNA activity and the orthogonality of a few orthogonal systems were significantly enhanced in a rapid and systematic manner towards their simultaneous use in *E. coli* [17]. Creation of a new tRNA identity may be achieved by introducing new tertiary structural elements [24,25] rather than by introducing small local modifications to tRNA. However, tRNA variants having an abnormal tertiary structure may be poorly compatible with the translation apparatus [8,16,26,27]. Thus, introduction of a small hairpin aptamer as the V-arm of orthogonal tRNAs may be a good solution for developing tRNA variants which are translationally active and have a characteristic tertiary structural element.

Recently, a new group of tRNAs, (9/3) allo-tRNAs, was identified in the raw short-read data of soil or marine metagenomic datasets [26,28]. These tRNAs have a non-canonical composition of the amino-acid acceptor branch (Figure 1A,B). While canonical tRNAs have a 7-bp accepter stem and a 5-bp TψC stem, the (9/3) allo-tRNAs have a 9-bp accepter stem and a 3-bp TψC stem (Figure 1A,B). In addition, the (9/3) allo-tRNAs have a long D-stem, like selenocysteine (Sec) tRNA species, and a long V-arm, like serine (Ser) and Sec tRNA species. Thus, the (9/3) allo-tRNAs intrinsically have identity elements for the Ser-tRNA synthetase (SerRS) and the Sec-tRNA synthesis apparatus [26,28,29]. Amber suppressor variants of two of the (9/3) allo-tRNA species were used for the site-specific Ser or Sec incorporation into proteins in response to the TAG amber stop codon in *E. coli* [29]. Two of the natural allo-tRNA species also have the essential identity elements for alanyl-tRNA synthetase (AlaRS) [26,29], and one of the two preferentially worked as tRNA^Ala^ rather than as tRNA^Ser^ in *E. coli* cells [26]. Despite the non-canonical secondary structure, many allo-tRNA species were highly compatible with the *E. coli* translation system and worked as exceedingly active nonsense or missense suppressor tRNAs in *E. coli* [26,29]. These findings prompted us to employ these (9/3) allo-tRNA scaffolds as tRNA chassis for making our tRNA-aptamer fusions. However, the strong intrinsic affinity of (9/3) allo-tRNA species for SerRS has limited their utility.

In the present study, a few kinds of SerRS-rejecting allo-tRNA chassis were developed. Several kinds of small hairpin aptamers inserted into these chassis were confirmed to be bound by their cognate proteins or protein domains expressed in *E. coli*. The new tRNA chassis, or “orthogonal allo-tRNAs”, will help to greatly expand the design flexibility of new tRNA-aaRS pairs.

## 2. Results

### 2.1. Strategy for tRNA Chassis Development

In an attempt to develop a SerRS-rejecting (9/3) allo-tRNA^Ser^ variant, we realized that certain allo-tRNA species (allo-tRNA^UTu2^ series [29], see Figure 1B) having a G:U wobble base pair at the bottom of the V-stem worked as less active tRNA^Ser^ than allo-tRNA variants carrying Watson-Crick (WC) changes (data not shown). It is known that a G:U pair in an RNA double helix introduces a helical twist [30] and might cause steric hindrance against target proteins [31,32]. Thus, it was suggested that the original allo-tRNA^UTu2^ variant (or S001 variant) may have a narrow (but enough) space between the TψC arm and the V-arm for the insertion of the *N*-terminal domain (NTD) of SerRS (Figure 1A) [33]. To verify this idea, we inserted a bulged uracil (U) nucleotide into the V-stem of the S001 variant to make S002 (Figure 1A,B). In a preliminary experiment, the S002 variant hardly incorporated serine into proteins in *E. coli*, meaning that S002 is either SerRS-rejecting or simply unstable (Figure 1). On the other hand, the Ala-accepting variant of S002, or A002, which has the major identity elements for AlaRS (G3:U70 with A73) [26,29,34], incorporated alanine into proteins (data not shown here) (Figure 1C). Thus, the S002/A002 scaffold may retain the tertiary structural stability but may not have enough space for accommodating the NTD of SerRS (Figure 1). Encouraged by these preliminary results, I decided to examine seventy-nine kinds of bulge-carrying variants of nine kinds of amber suppressor (9/3) allo-tRNA species (Figure 1B and Figure S1) [26] in order to search for better chassis tRNAs than the S002 variant. Experiments were facilitated by use of *E. coli* cells with several reporter genes carrying an in-frame TAG codon (Figure 1C).

### 2.2. Searching for Candidate tRNA Chassis

In this study, all tRNA sequences were cloned in a high-copy-number plasmid vector under control of the arabinose promoter [26,29]. The starting amber suppressor allo-tRNA variants (named as S001, S012, S020, S026, S032, S043, S047, S051, and S055) were developed from nine allo-tRNA species (Figure 1B and Figure S1) chosen from ten allo-tRNA species found in nature [26]. Positional effects of single bulge nucleotide or two separate or successive bulge nucleotides on the V-arms of these allo-tRNA scaffolds were investigated by individually examining seventy-nine kinds of bulge-carrying variants (see supplementary Excel file). Their “Ala-acceptable” variants carrying the major identity elements for AlaRS (G3:U70 with A73) were also developed, although other structural elements contribute to the tRNA recognition by AlaRS [34,35]. A chloramphenicol acetyltransferase (CAT) reporter gene in which the Ser146 codon had been mutated to TAG, or *cat(Ser146TAG)* [26], was used for examining the TAG-translating activities of these tRNA variants in a suppressor-free strain of *E. coli* DH10B. While the replacement of Ser146 by Ala results in a partially active enzyme, replacement of Ser146 by bulkier residues than Ser resulted in loss of function [26,36]. Consequently, the *cat(Ser146TAG)* reporter is useful for detecting a strong Ala-inserting activity and even a weak Ser-inserting activity of allo-tRNAs in chloramphenicol (Cm)-sensitive *E. coli* (Figure 1C) [26]. Two conditions for a good candidate allo-tRNA chassis were that the Ala-acceptable variant inserts Ala, while the variant lacking the G3:U70 and A73 elements hardly inserts Ser (Figure 1C).

**Figure 2:**
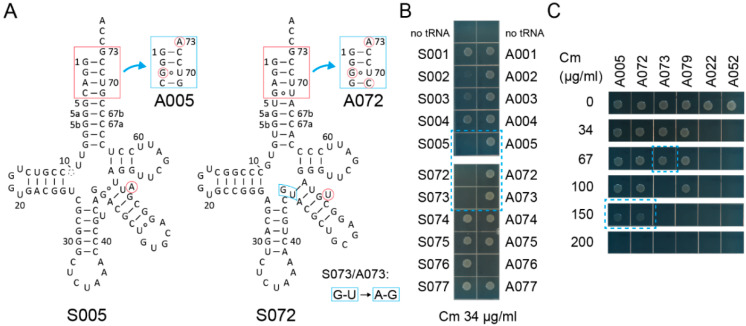
Candidate tRNA chassis. (**A**) The cloverleaf structures of the S005 and S072 tRNA variants and their Ala-accepting variants (A005 and A072, respectively). The S073/A073 variants have two indicated modifications compared to the S072/A072 variants, respectively. (**B**) The S005, S072, and S073 variants conferred lower chloramphenicol (Cm) resistance (less than Cm 34 µg/mL) to *E. coli* DH10B cells expressing the *cat(Ser146TAG)* reporter gene compared to the S002 variant. (**C**) The A005 and A072 variants conferred up to Cm 150 µg/mL resistance to cells, while the A073 variant conferred only Cm 67 µg/mL resistance.

By comparing 78 variant pairs to the S002/A002 pair, S005/A005, S072/A072, and S073/A073 pairs (Figure 2A) passed the first screening using the *cat(Ser146TAG)* gene with Cm at a concentration of 34 µg/mL in the growth media (Figure 2B and Table S1). While S002 conferred marginal Cm resistance to cells, S005, S072, and S073 hardly conferred Cm resistance to cells (Figure 2B). A005 and A072 conferred up to 150 µg/mL Cm resistance, while A073 conferred only up to 67 µg/mL Cm resistance (Figure 2C). The S002/A002 and S005/A005 variant pairs are both derived from S001/A001 and have a bulged U or A, respectively, at different positions in the V-stem (Figure 2A). It is likely that S005 is more SerRS-rejective than S002. Because the S072/A072 and S073/A073 variant pairs differ only at the junction of the anticodon stem and the V-stem (Figure 2B), the A073 variant may be either less stable or less compatible to AlaRS than the A072 variant. Whereas the S079/A079 pair derived from S043 also passed the first screening (Table S1, Figure 2C), the other S043-derived variants retained their SerRS identity (Table S1) possibly due to the G20 residue (Figure S1). Thus, I assumed that the S005/A005 and S072/A072 chassis may be the most reliable ones.

### 2.3. Sequestration of Aptamer-tRNA Fusion RNAs

**Figure 3:**
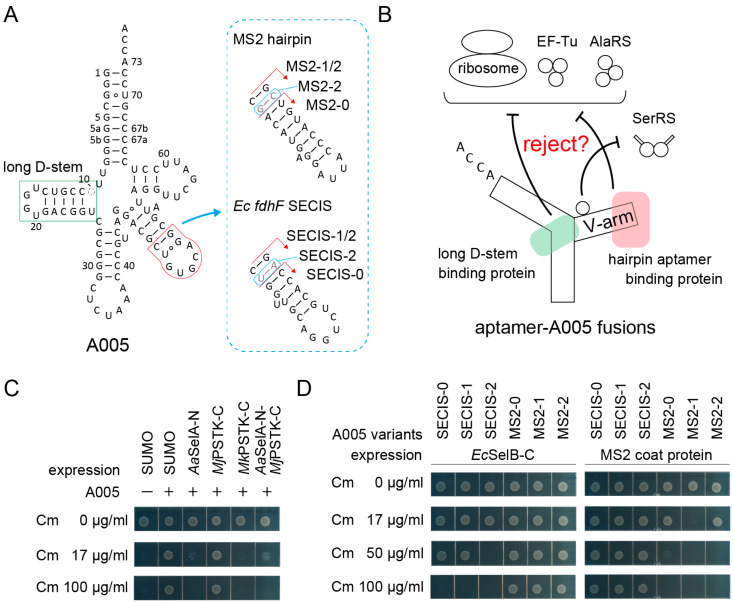
Sequestration of aptamer-tRNA fusions from the *E. coli* translation system via the expression of the cognate aptamer-binding proteins. (**A**) The cloverleaf structure of A005 tRNA variants having a hairpin RNA aptamer in the V-arm. The long D-stem of (9/3) allo-tRNAs and tRNA^Sec^ species is a binding site for tRNA^Sec^-binding enzymes SelA and PSTK. (**B**) Strategy for the sequestration of the aptamer-A005 fusion variants. (**C**) The A005 tRNA molecules were almost fully sequestered by the *N*-terminal domain of *Aquifex aeolicus* (*Aa*) SelA and the *C*-terminal domain of *Methanopyrus kandleri* (*Mk*) PSTK but not by the *C*-terminal domain of *Methanocaldococcus jannaschii* (*Mj*) PSTK. (**D**) The SECIS-A005 fusions were sequestered by the *C*-terminal domain of *E. coli* SelB, while the MS2-A005 fusions were sequestered by the MS2 coat protein. Among the fusion tRNAs, the A005-MS2-1 variant was most effectively sequestered. The SUMO protein was used as a negative control and as a *N*-terminal solubilizing tag for the SelA, PSTK, and SelB domains.

Tight protection of an aptamer moiety by its cognate protein will result in sequestration of the aptamer-tagged allo-tRNA molecules from the translation apparatus (Figure 3A,B). Previously, we observed that (9/3) allo-tRNA molecules were fully sequestered when selenocysteine synthase (SelA) molecules were overexpressed [29]. As shown in Figure 3A, (9/3) allo-tRNAs have a long D-stem which is specifically recognized by the *N*-terminal domain of SelA (SelA-N) [37] and the *C*-terminal domain of archaeal *O*-phosphoseryl-tRNA kinase (PSTK-C) [38]. As expected, the Ala-inserting activity of the A005 variant was almost fully removed when the *Aquifex aeolicus* (*Aa*) SelA-N or the *Methanopyrus kandleri* (*Mk*) PSTK-C were expressed at a moderate level by using the *tac* promoter on a pGEX vector and IPTG at a concentration of 10 µM (Figure 3C). In the presence of these proteins, A005 hardly conferred 17 µg/mL Cm resistance to cells (Figure 3C), which demonstrated that this reporter assay system is valid. Interestingly, the *Methanocaldococcus jannaschii* (*Mj*) PSTK-C failed to sequester A005 tRNA molecules (Figure 3C), possibly because *Mj* tRNA^Sec^ has a longer D-stem than those of *Mk* tRNA^Sec^ and allo-tRNAs [38]. Similar results were obtained by using the A072 variant (data not shown). Next, the V-arm moiety was engineered to mediate the sequestration of tRNA molecules. Two kinds of small hairpin aptamers, the MS2 hairpin RNA [9,11] and the *E. coli fdhF* SECIS RNA [3], were transplanted to the A005 variant with varying lengths of adaptors (Figure 3A). The in vivo interaction of the transplanted MS2 RNA and SECIS RNA with a MS2 coat protein dimer [9,11] and the *C*-terminal WH3/4 domains of *E. coli* SelB [3], respectively, were examined. As expected, the A005-MS2 variants were sequestered by the coat protein, while the A005-SECIS variants were sequestered by the WH3/4 domains (Figure 3D). Among the three A005-MS2 variants, A005-MS2-1 with the medium-length adapter was most efficiently sequestered by MS2 coat protein (less than 17 µg/mL Cm resistance) (Figure 3D). Among the three A005-SECIS variants, A005-SECIS-2 with the longest adapter was most efficiently sequestered by the WH3/4 domains (up to 17 µg/mL Cm resistance) (Figure 3D). Thus, the optimal length of the adapter stem varied for each aptamer-protein pair. These experiments demonstrated that a small hairpin aptamer can be safely installed into an allo-tRNA chassis without significantly impairing the tRNA function in the absence of the target protein.

**Figure 4:**
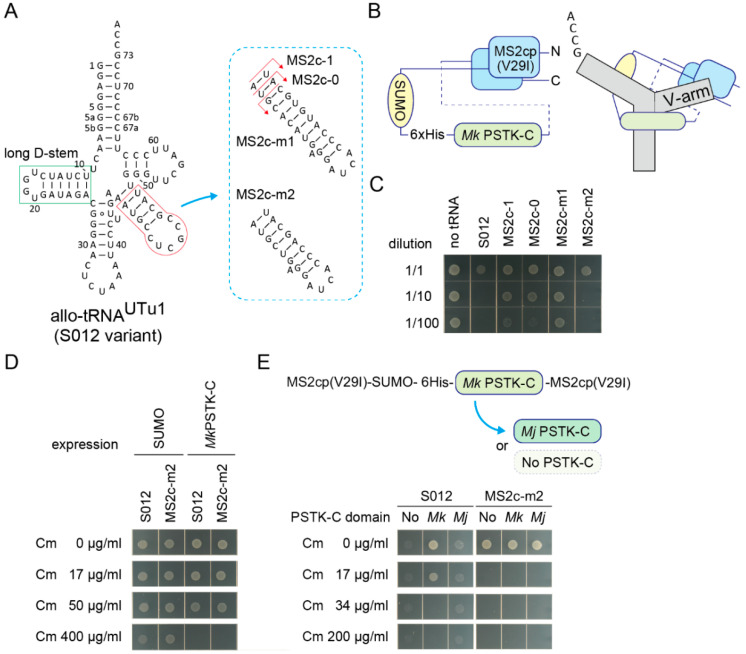
Sequestration of fully active allo-tRNA^Ser^ molecules. (**A**) The cloverleaf structure of S012 tRNA variants having a MS2 hairpin *C*-loop variant with varying lengths of adaptors. The long D-stem of S012 variants is tightly bound by the *C*-terminal domain of *Mk* PSTK. (**B**) A structural model of the fusion protein consisting of an MS2 coat protein V29I variant monomer, a SUMO tag, a 6xHis tag, a *Mk* PSTK-C, and another V29I monomer. The *N*-terminus and *C*-terminus are indicated. A binding model of a S012 variant and this fusion protein is shown, too. (**C**) The S012 and MS2c-m2 variants most efficiently impaired the growth of *E. coli* DH10B cells expressing the *cat(Ser146TAG)* reporter gene and the SUMO protein. Cell cultures were diluted (10 times and 100 times) or non-diluted and spotted on an agar plate. The arabinose concentration was raised to 0.1% from 0.01% to observe clear differences. (**D**) The *Mk* PSTK-C protein not fused with the MS2 coat protein failed to fully sequester both S012 and MS2c-m2 molecules in *E. coli* DH10B cells expressing the *cat(Ser146TAG)*. (**E**) The *Mk* PSTK-C domain of the chimeric fusion protein was replaced with an *Mj* PSTK-C domain or simply removed. All fusion protein variants perfectly sequestered MS2c-m2 molecules in *E. coli* DH10B cells expressing the *cat(Ser146TAG)*, indicating that the single-chain coat protein moiety tightly bound with the MS2 hairpin moiety of MS2c-m2. On the other hand, S012 molecules were sequestered only by the *Mk* PSTK-C-containing fusion protein. The other fusion proteins failed to repress the toxicity and the suppressor tRNA activity of the S012 molecules. Note that cells are unclear but were growing on the Cm-free and Cm-containing agar plates under the S012 + No/Mj conditions.

Next, sequestration of fully active tRNA^Ser^ molecules was challenged. It is known that the allo-tRNA^UTu1^ variant (Figure 4A and Figure S2A) [29] (or S012 in this study) is as active a tRNA^Ser^ as one of the *E. coli* tRNA^Ser^ species in *E. coli*. S012 conferred at least 600 µg/mL Cm resistance to *E. coli* expressing the *cat(Ser146TAG)* gene (Figure S2B) and at least 34 µg/mL Cm resistance to cells additionally expressing the *Mk* PSTK-C (Figure S2C,D). Thus, full sequestration of these fully active tRNA molecules may require another tight interaction between an RNA structure and its target protein. Instead of the wildtype MS2 hairpin RNA and the wildtype MS2 coat protein, an MS2 hairpin RNA variant with a *C*-loop (U to C at the -5 position) [39,40] and the MS2 coat protein V29I variant [40,41] were used to enhance the RNA aptamer-coat protein interaction. To make a single-chain MS2 coat protein [42] together with an *Mk* PSTK-C domain, two V29I domains were fused with a long linker containing a SUMO domain, a 6xHis tag, and an *Mk* PSTK-C domain, in this order (Figure 4B). This chimeric fusion protein efficiently sequestered a S012 variant having an MS2 *C*-loop hairpin with a 1-bp adaptor, or S012-MS2c-1, in *E. coli* expressing the *cat(Ser146TAG)* gene (Figure 4A and Figure S3). However, it seems likely that the S012-MS2c-1 variant was less active than the S012 variant, as the original S012 variant was exceedingly toxic to cells (Figure 4C), probably due to its high suppression efficiency [26,29]. Thus, the V-arm adaptor length of the S012-MS2c-1 variant was optimized to restore its tRNA activity in the absence of the chimeric protein (Figure 4A). One variant that has the smallest V-arm (S012-MS2c-m2) showed a similar toxicity to the S012 variant (Figure 4A,C). Both the S012 and S012-MS2c-m2 variants still conferred at least 50 µg/mL Cm resistance to *E. coli* expressing the *cat(Ser146TAG)* gene and the *Mk* PSTK-C domain not fused with the MS2 coat protein (Figure 4D). Then, the sequestration efficiencies of S012 and S012-MS2c-m2 molecules by the chimeric fusion protein were examined (Figure 4E). Surprisingly, expression of the chimeric fusion protein completely repressed the Ser-inserting activities of S012-MS2c-m2 molecules (Figure 4E), while S012 molecules were not completely sequestered. To see the contribution of the *Mk* PSTK-C domain moiety and the single-chain coat protein moiety in the chimeric fusion protein, the *Mk* PSTK-C domain was removed or replaced by an *Mj* PSTK-C domain which did not sequester allo-tRNAs (Figure 4E). All fusion proteins sequestered S012-MS2c-m2 molecules in a complete manner (Figure 4E). Thus, the single-chain coat protein moiety may have bound to the MS2 hairpin moiety of the S012-MS2c-m2 molecules and inhibited their interaction with SerRS. In contrast, the partial sequestration of S012 molecules was dependent on the *Mk* PSTK-C domain (Figure 4E). These results demonstrated that the strong protein-RNA interaction led to the complete sequestration of allo-tRNA molecules.

### 2.4. Synthetic Tyrosine tRNAs

**Figure 5:**
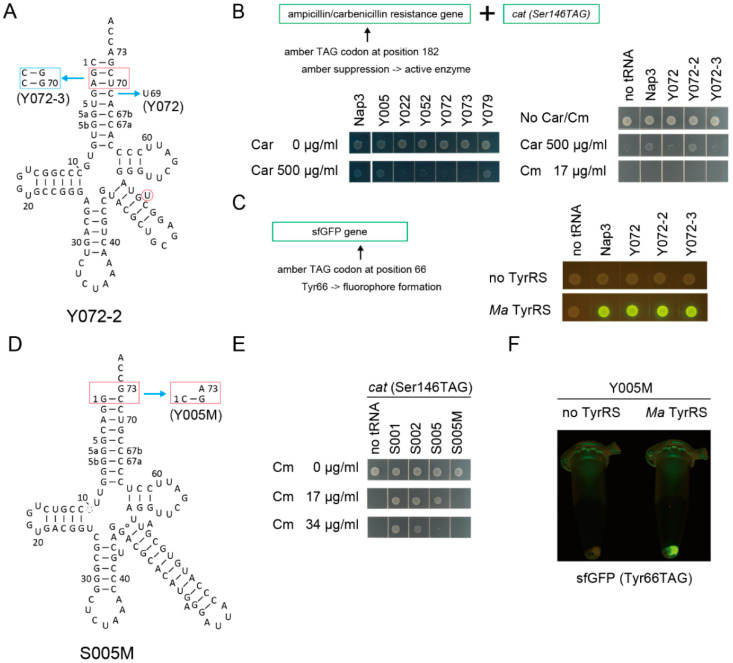
Development of synthetic tyrosine tRNAs using candidate tRNA chassis. (**A**) The cloverleaf structure of Y072-series tRNA variants. (**B**) The S072 and S073 variants conferred only marginal carbenicillin (Car) resistance (less than Car 500 µg/mL) to *E. coli* DH10B cells expressing the *bla(Ala182TAG)* reporter gene and the *cat(Ser146TAG)* reporter gene in the absence of any heterologous TyrRS enzyme. Amber suppressor variants of archaeal *M. jannaschii* tRNA^Tyr^ species (Nap3) and the Y005, Y079, and Y072-2 variants conferred up to 500 µg/mL Car resistance, indicating that these tRNAs were to a less extent charged by endogenous aaRSs. No Cm resistance was introduced by the Y072, Y072-2, and Y072-3 variants, indicating that they were orthogonal to SerRS. (**C**) In the presence of Ca. M. alvus TyrRS in *E. coli* DH10B, the Nap3 variant and the three Y072-series variants translated the amber codon in the *sfGFP(Tyr66TAG)* reporter gene with tyrosine, making the fluorophore of sfGFP. (**D**) The cloverleaf structure of the S005M and Y005M tRNA variants. (**E**) The S005M variant conferred a lower Cm resistance (less than Cm 17 µg/mL) to *E. coli* DH10B cells expressing the *cat(Ser146TAG)* reporter gene compared to the S002 and S005 variants. (**F**) In the presence of Ca. M. alvus TyrRS in *E. coli* DH10B, the Y005M variant translated the amber codon of the *sfGFP(Tyr66TAG)* reporter gene with tyrosine, making the fluorophore of sfGFP.

Can the candidate tRNA chassis gain any amino-acid identity other than alanine and serine? To confirm the utility of the candidate tRNA chassis, the major identity elements for the archaeal-type tyrosyl-tRNA synthetase (TyrRS) (C1:G72 with A73) [43,44,45] were installed in the candidate tRNA chassis (Figure 5A), in order to develop synthetic Tyr tRNA molecules [44]. Y005, Y072, and Y073 variants were developed from S005, S072, and S073, respectively. Two derivatives of Y072 (Y072-2 and Y072-3) were also developed because modifications of the second and third base pairs in the acceptor stem may affect aminoacylation (see Figure 5A). An amber suppressor variant of the tRNA^Tyr^ species of *Methanocaldococcus jannaschii* (*Mj*), or Nap3 [46], was used as a positive control. An archaeal TyrRS species from *Candidatus* Methanomethylophilus alvus Mx1201 (*Ma*) was used to be paired with these tRNAs. Three kinds of reporter genes were used for examining the orthogonality and the tRNA^Tyr^ activity of these tRNA variants (Figure 5B,C). One is an ampicillin/carbenicillin resistance gene (*bla*) having an in-frame TAG codon at a permissive site (Ala182) [47] (Figure 5B). The *cat(Ser146TAG)* reporter gene was cloned together with this *bla(Ala182TAG)* reporter gene to make a dual-reporter plasmid. The third one is a sfGFP gene in which the Tyr66 codon had been mutated to TAG, since Tyr66 is essential for the fluorophore formation [48] (Figure 5C). Using the *bla* reporter gene, it was revealed that the Y072 and Y073 variants conferred only marginal carbenicillin (Car) resistance to cells, while the Y005 variant and the *Mj* tRNA^Tyr^ Nap3 variant conferred up to 500 µg/mL Car resistance to cells (Figure 5B). Note that the Nap3 variant has been used as a representative orthogonal tRNA^Tyr^ in *E. coli* [49]. This result indicated that the Y072 and Y073 variants were not activated by any endogenous aaRS species in *E. coli*. Although the Y072-2 variant conferred up to 500 µg/mL Car resistance to cells (Figure 5B), none of the Y072, Y072-2, and Y072-3 variants conferred Cm resistance to *E. coli* DH10B cells expressing the *cat(Ser146TAG)* reporter gene (Figure 5B). Thus, these variants showed no Ser identity. Next, by using the sfGFP(Tyr66TAG) reporter gene, it was confirmed that the Y072, Y072-2, and Y072-3 variants were charged with tyrosine by *Ma* TyrRS (Figure 5C).

To improve the usefulness of the S005 chassis, I examined another chassis, S005M, which is a variant of A005-MS2-1 lacking the AlaRS identity elements (Figure 5D). It was found that the original S005 variant conferred 17 µg/mL Cm resistance to *E. coli* DH10B cells expressing the *cat(Ser146TAG)* reporter gene (Figure 5E). In contrast, the S005M variant having an MS2 hairpin at the tip of the V-arm did not confer 17 µg/mL Cm resistance (Figure 5E). Most likely, the S005M variant may have a stabilized bulge structure and a slightly reduced tRNA activity compared to S005. Although the marginal Ser-inserting activity of S005 can be eliminated by engineering the tip of the acceptor stem [22], the use of S005M facilitates engineering of the amino-acid identity owing to the ignorable Ser-inserting activity. The S005M chassis was engineered to be coupled with *Ma* TyrRS, to develop Y005M (Figure 5D). In the presence of *Ma* TyrRS, Y005M translated the amber codon of the sfGFP(Tyr66TAG) reporter gene with tyrosine (Figure 5F). These results clearly demonstrated that SerRS-rejecting allo-tRNA chassis can be engineered and converted to synthetic Tyr tRNA species.

### 2.5. Enhancing Aminoacylation by Aptamer-Protein Interaction

**Figure 6:**
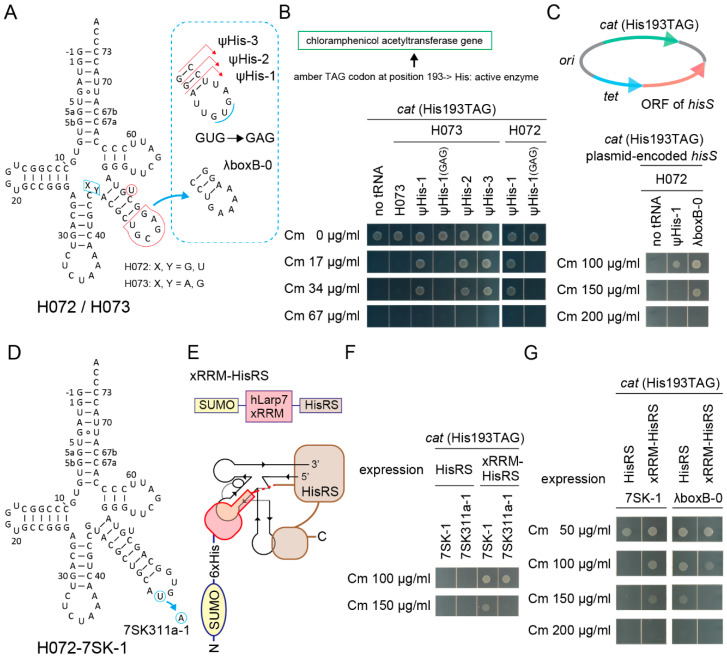
Development of synthetic histidine tRNAs using candidate tRNA chassis and hairpin aptamers. (**A**) The cloverleaf structures of H072 and H073 tRNA variants having an extended V-stem and a modified V-arm loop sequence. (**B**) The H073 ψHis-1/2/3 variants and the H072 ψHis-1 variant conferred up to Cm 34 µg/mL resistance to *E. coli* DH10B cells expressing the *cat(His193TAG)* reporter gene. The catalytic His193 residue is essential for the Cm acetyltransferase activity. A point mutation in the V-arm loop sequence (from GUG to GAG) eliminated the His-inserting activity of the ψHis-1 variants. (**C**) The H072 ψHis-1 and λboxB-0 variants conferred up to Cm 100 µg/mL resistance and 150 µg/mL resistance, respectively, to *E. coli* DH10B cells expressing the *cat(His193TAG)* reporter gene and the plasmid-encoded *hisS* gene (*E. coli* HisRS) shown. Note that the agarose plate containing Cm 100 µg/mL was incubated for another four hours to obtain the clear cell spot for H072 ψHis-1. (**D**) The cloverleaf structure of the H072-7SK-1 variant having a 7SK SL4 hairpin. (**E**) A chimeric *E. coli* HisRS variant fused with the xRRM domain of human Larp7. A binding model of H072-7SK-1 and this xRRM-HisRS is shown. The dashed line indicates linker residues SSGSNSNSGS. (**F**) Expression of xRRM-HisRS enhanced the His-inserting activities by the H072-7SK-1 and H072-7SK311a-1 variants. The H072-7SK-1 variant conferred up to 150 µg/mL resistance to *E. coli* DH10B cells expressing the *cat(His193TAG)* reporter gene and xRRM-HisRS. It was reported that the 7SK RNA SL4 binds the hLarp7 xRRM domain, while the 311a variant binds the xRRM domain more tightly. (**G**) The His-inserting activity of H072-7SK-1 was enhanced by xRRM-HisRS, while that of H072-λboxB-0 was not enhanced.

Can an RNA aptamer moiety in tRNA work as an additional binding site for an aaRS fused with the aptamer-binding protein domain? To answer this, synthetic histidine (His) tRNA molecules [18] were developed by installing the major identity element for the *E. coli* histidyl-tRNA synthetase (HisRS) (G-1:C73) [2,26,50] into the S072/S073 chassis (Figure 6A). Two kinds of *cat* reporter genes, *cat(Ser146TAG)* and *cat(His193TAG)*, were used for examining the orthogonality and the His-inserting activity of designed tRNA variants. In the *cat(His193TAG)* gene, the essential catalytic His193 was changed to TAG [51]. It soon turned out that a S073 variant with a G-1:C73 pair (named H073) was not charged by the endogenous HisRS in *E. coli* expressing the *cat(His193TAG)* gene (Figure 6B). However, extending the V-stem length and modifying the V-arm loop sequence produced functional H072/H073 variants which conferred up to 34 µg/mL Cm resistance to cells (Figure 6B). Ser insertion was not mediated by these H072/H073 variants in *E. coli* cells expressing the *cat(Ser146TAG)* gene (Figure S4A). Point mutations in the V-arm loop sequence affected the His-inserting activity of H072 ψHis-1 (Figure 6A); UUUUGAU was better than the original UUGUGAU, while UUGAGAU and UUGCGAU were less suitable (Figure 6B and Figure S4B,C), implying that a good V-arm loop sequence on a moderately long V-arm may be rich in uracil residues. It was also found that a moderate overexpression of HisRS from a plasmid-encoded *hisS* gene enhanced the His-inserting activities by the synthetic His tRNAs (Figure 6C and Figure S4F). Up to 150 µg/mL Cm resistance was conferred by the H072-λboxB-0 variant (Figure 6A,C). On the other hand, the S005 chassis was useless for developing a synthetic His tRNA in the same manner (Figure S4D,E). Thus, it was revealed that the H072/H073 chassis were intrinsically compatible to *E. coli* HisRS and *E. coli* RNase P, which cleaves His tRNA precursors at the -1 position [52].

By using the H072 chassis and *E. coli* HisRS, several cognate pairs of RNA aptamers and protein domains [2,28,50,53,54,55] were examined (Figure S5A,B). The first preliminary experiment revealed that the cognate pair of the 7SK RNA stem-loop 4 (SL4) and the hLarp7 xRRM domain [53] fused to the *N*-terminus of *E. coli* HisRS (Figure 6D,E) might have enhanced the His-inserting activity by the tRNA-aptamer fusion in *E. coli* expressing the *cat(His193TAG)* gene (Figure S5C). The other pairs examined seemed to be rather inhibitory (Figure S5C). Three explanations are possible: 1) addition of an *N*-terminal domain might have reduced the expression level, stability, accessibility, or activity of the tethered HisRS, 2) too strong affinity between the cognate RNA-protein pair might have reduced the turnover rate of aminoacylation, and 3) a too short linker sequence SSGSNSNSGS between the *N*-terminal domain and HisRS might have kept the catalytic pocket of the tethered HisRS away from the CCA tail of tRNA. In contrast, the xRRM domain binds a 7SK SL4 RNA with a moderate affinity (Kd = 129 nM [53]). Furthermore, the *C*-terminus of the xRRM domain and the *N*-terminus of the *E. coli* HisRS may approach each other on the tRNA and can be fused by using the short linker (Figure 6E), according to crystal structural studies [50,53]. In another experiment, the H072-7SK-1 variant conferred up to 150 µg/mL resistance to *E. coli* DH10B cells expressing the *cat(His193TAG)* reporter gene and xRRM-HisRS (Figure 6F), while the U311A change in the 7SK SL4 loop (Figure 6D), which reportedly enhances the interaction (Kd = 51 nM [53]), slightly reduced the His-inserting activity (Figure 6F). The U311A change may have slightly impaired the turnover of the enzyme or slightly destabilized the tRNA. Lastly, the effect of the xRRM domain on aminoacylation was confirmed by using the H072-7SK-1 and H072-λboxB-0 (Figure 6A) variants. The expression of xRRM-HisRS greatly enhanced the His-inserting activity of H072-7SK-1 (Figure 6G), while H072-λboxB-0 was efficiently activated by the wildtype HisRS but not by xRRM-HisRS (Figure 6G). This meant that the appended SUMO-xRRM domains were rather inhibitory to histidylation in the absence of the cognate interaction between the xRRM domain and the aptamer moiety, probably due to the decreased expression level, stability, or accessibility of HisRS. Thus, it was concluded that the cognate interaction contributed to the enhancement of aminoacylation.

### 2.6. Recruiting aaRS via a Pseudo-Anticodon in the V-Arm

**Figure 7:**
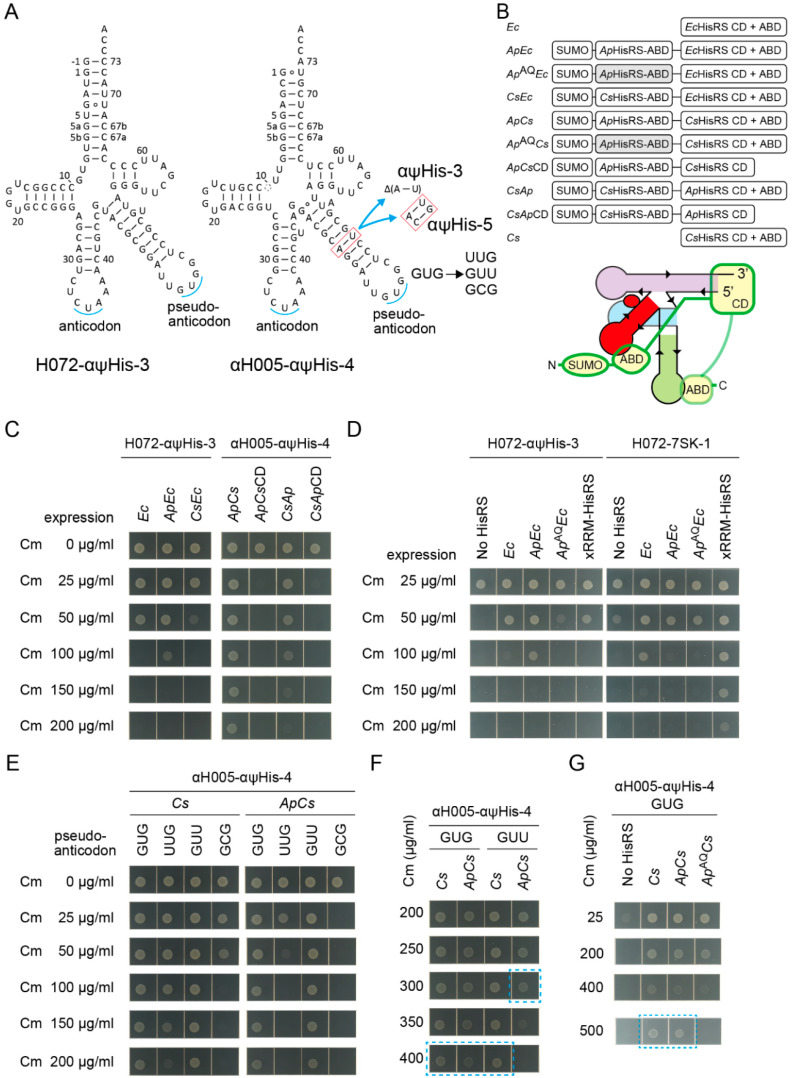
Recruiting HisRS via a pseudo-anticodon arm. (**A**) The cloverleaf structures of the H072-αψHis-3 and αH005-αψHis-3/4/5 variants with varying lengths of adaptors and with diverse pseudo-anticodon sequences. (**B**) The domain structures of HisRS and HisRS variants *N*-terminally fused with a heterologous anticodon binding domain (ABD). CD denotes catalytic domain. *Ec*, *Ap*, and *Cs* denote *E. coli*, *Afifella pfennigii*, and *Consotaella salsifontis*, respectively. *Ap*^AQ^ denotes an *Ap* ABD variant carrying E484A-K493Q mutations impairing anticodon recognition. (**C**,**D**) Cm resistance of *E. coli* DH10B (NEB 10-beta) cells expressing the *cat(His193TAG)* reporter gene and the indicated combinations of HisRS and tRNA variants. (**D**) The His-inserting activity of H072-αψHis-3 was enhanced by *ApEc* but not enhanced by *Ap*^AQ^*Ec*, indicating that the recognition of the GUG pseudo-anticodon by the wildtype *Ap* ABD moiety was important. On the other hand, the His-inserting activity of H072-7SK-1 lacking the GUG pseudo-anticodon was not enhanced by both *ApEc* and *Ap*^AQ^*Ec.* No HisRS indicates that only the genomic *hisS* gene was expressing. (**E**–**G**) Cm resistance of *E. coli* DH10B (NEB 10-beta) cells expressing the *cat(His193TAG)* reporter gene and the indicated combinations of HisRS variants and pseudo-anticodon variants of αH005-αψHis-4. The Cm concentrations are indicated. (**G**) Note that the agarose plate containing Cm 500 µg/mL was incubated for another four hours to obtain clear cell spots.

Can an aaRS variant having an additional heterologous anticodon binding domain (ABD) be recruited to tRNA via the V-arm which mimics the anticodon arm that can be recognized by the ABD? In other words, can a pseudo-anticodon arm moiety [1,56] on tRNAs work as an aptamer for the ABD and contribute to aminoacylation enhancement? Here, the full-length HisRSs, HisRS catalytic domains (CDs), and HisRS ABDs of two α-Proteobacteria species and their tRNA^His^ anticodon arm were used. It is well known that a large subgroup of α-Proteobacteria has a non-canonical tRNA^His^ lacking the G-1:C73 pair and has a non-canonical HisRS species that recognizes the non-canonical G1:U72 pair and A73 in addition to the GUG anticodon [17,57,58,59,60]. The two α-proteobacterial species are *Afifella pfennigii* DSM 17,143 (*Ap*) [17] and *Consotaella salsifontis* USBA 369 (*Cs*) [61], because the well-studied α-proteobacterial *Caulobacter crescentus* HisRS was not active enough in *E. coli* [60]. Two kinds of allo-tRNA chassis, S072 and S005, were engineered to have a typical α-proteobacterial tRNA^His^ anticodon arm as their V-arms, to develop H072-αψHis-3 and αH005-αψHis-3/4/5 variants (Figure 7A). The H072-αψHis-3 variant has the G-1:C73 element to be paired with *E. coli* HisRS variants *N*-terminally fused with an α-proteobacterial ABD using a 20 amino-acid linker (Figure 7B). On the other hand, the αH005-αψHis-3/4/5 variants have the G1:U72 and A73 elements to be paired with *Ap/Cs* HisRS *N*-terminally fused with a heterologous ABD using the same 20 amino-acid linker (Figure 7B). The *C*-terminal ABDs of chimeric Ap/Cs HisRS variants were kept intact or trimmed (Figure 7B). *ApEc*, *CsEc*, *ApCs*, and *CsAp* variants denote *Ap*ABD-*Ec*HisRS, *Cs*ABD-*Ec*HisRS, *Ap*ABD-*Cs*HisRS, and *Cs*ABD-*Ap*HisRS, respectively (Figure 7B).

By using the *cat(His193TAG)* reporter system, the His-inserting activities by combinations of the tRNA variants and HisRS variants were examined (Figure 7C). Addition of the *N*-terminal *Ap*ABD, but not *Cs*ABD, to the *E. coli* HisRS led to the enhancement of the His-inserting activity by H072-αψHis-3 (Figure 7C). Thus, the *Ap*ABD may be especially useful in *E. coli*, as explicitly predicted in a previous study [17]. Next, the αH005-αψHis-3/4/5 variants were combined with the chimeric *Ap/Cs* HisRS variants. The *ApCs* variant efficiently activated the αH005-αψHis-3/4/5 variants, while the *CsAp* variant was less active (Figure 7C and Figure S6). Among the αH005-αψHis-3/4/5 variants, αH005-αψHis-4 seems the most active, because αH005-αψHis-3 and αH005-αψHis-5 conferred slightly lower Cm resistance (Figure S6). In contrast, the chimeric *Ap/Cs* HisRS variants lacking the original *C*-terminal ABD were totally inactive (Figure 7C), probably because HisRS ABD is responsible not only for anticodon recognition but also for HisRS dimerization and for precise acceptor stem discrimination [18,62]. Thus, it is evident that the tRNA variants having a GUG pseudo-anticodon arm were charged with histidine by HisRS variants *N*-terminally fused with an *Ap*ABD.

To confirm the involvement of the GUG pseudo-anticodon in the tRNA-HisRS interaction, two lines of experiments were performed. First, the H072-αψHis-3 variant (having the GUG pseudo-anticodon) and the H072-7SK-1 variant (having no pseudo-anticodon arm) were compared (Figure 7D). To partially impair the anticodon recognition activity of the *Ap* ABD, two mutations (E484A-K493Q) which may partially impair anticodon recognition [50] were introduced to develop *Ap*^AQ^*Ec* and *Ap*^AQ^*Cs* HisRS variants (Figure 7B). As expected, *ApEc* enhanced the histidylation of H072-αψHis-3, whereas *Ap*^AQ^*Ec* failed (Figure 7D). H072-αψHis-3 was activated at similar efficiencies by wildtype HisRS and xRRM-HisRS (Figure 7D). In contrast, the histidylation of H072-7SK-1 was significantly enhanced by xRRM-HisRS (Figure 6G,7D). Both *ApEc* and *Ap*^AQ^*Ec* variants failed to enhance the histidylation of H072-7SK-1 (Figure 7D). Thus, it was revealed that the interaction between the GUG pseudo-anticodon arm and the *Ap* ABD moieties contributed to the enhancement of aminoacylation. However, compared to the 7SK-xRRM interaction (Figure 6E), this GUG-*Ap*ABD interaction exerted a modest effect on aminoacylation enhancement (Figure 7D).

The second line of experiments were performed to examine the importance of the GUG pseudo-anticodon sequence. The GUG sequence in αH005-αψHis-4 was changed to UUG, GUU, and GCG (Figure 7A). According to a previous study, *C. crescentus* tRNA^His^ variants having these anticodon changes are poor substrates of *C. crescentus* HisRS [58]. Surprisingly, the wildtype *Cs* HisRS charged all pseudo-anticodon variants of αH005-αψHis-4 (Figure 7E). Thus, it is evident that the critical recognition elements of tRNA^His^ for *Cs* HisRS are the G1:U72 pair with the A73 discriminator base but not the GUG anticodon. In the presence of *Cs* HisRS, the original GUG variant and the GUU variant conferred at least Cm 400 µg/mL resistance (Figure 7F), while the UUG and GCG variants conferred only up to Cm 150 µg/mL and Cm 50 µg/mL resistance, respectively (Figure 7E). In the presence of *Cs* HisRS or *ApCs*, the original GUG variant conferred up to Cm 500 µg/mL resistance, while it still conferred at least Cm 400 µg/mL resistance in the presence of *Ap*^AQ^*Cs* (Figure 7G). Thus, this GUG-*Ap*ABD interaction exerted only a modest effect on aminoacylation enhancement, probably because αH005-αψHis-4 is already a good substrate of wildtype *Cs* HisRS. In contrast, the UUG/GUU/GCG pseudo-anticodon variants were charged by *ApCs* much less efficiently than they were charged by *Cs* HisRS (Figure 7E,F). This indicated that the *N*-terminal appended domain was rather inhibitory to histidylation in the absence of the cognate interaction between the pseudo-anticodon and the appended ABD. In summary, it was shown that a pseudo-anticodon arm moiety can contribute to the enhancement of aminoacylation efficiency and tRNA-recognition specificity.

## 3. Discussion

In the present study, I showed that the SerRS-rejecting allo-tRNA chassis can be engineered to transfer Ala, Tyr, and His and fused with several kinds of hairpin RNAs. Although this study lacks any biochemical data, combinations of traditional in vivo reporter assay experiments clearly demonstrated that some of the RNA aptamers installed into the allo-tRNA chassis were bound by their cognate proteins or protein domains. Furthermore, the interactions between RNA aptamers and their cognate protein domains contributed to the enhancement of the tRNA-recognition specificity and the histidyl-tRNA formation by chimeric HisRS variants. Our next task should be simultaneous in vivo selection of the aptamer moieties and protein domains to optimize the V-stem length, the V-arm loop sequence, and the aptamer-recognizing residues of the protein domains. This optimization step may also be required for reducing the affinity of high-affinity aptamers and for preventing misrecognition of endogenous RNA molecules by the protein domains.

Some of the methods developed in this study may be applicable to genetic code expansion and genetic code reprogramming. For example, the pairs of Ca. M. alvus TyrRS and Y072/Y005M and the pairs of *Cs* HisRS variants and αH005-αψHis-4 variants may be orthogonal in *E. coli* and might be useful for incorporating non-canonical amino acids [17,58,60]. Alternatively, some of the endogenous tRNAs or tRNA-aaRS pairs might be replaceable with allo-tRNAs or orthogonal pairs composed of allo-tRNAs [60,63,64,65]. For reprogramming the genetic code, the allo-tRNA sequestration system may be useful for use in vivo and in vitro [66] and for the purification and elimination of allo-tRNA molecules from tRNA mixtures [66,67,68,69]. Application of the allo-tRNA chassis may not be limited to *E. coli* but may possibly need further optimization, especially for their use in archaea and eukaryotes due to the difference in the manner of tRNA^Ser^ recognition by SerRS [70,71].

In recent years, synthetic biologists have started to create synthetic tRNA-aminoacylation protein enzymes and ribozymes [18,72,73]. However, unlike most of the natural aaRS species, these artificial protein enzymes, including my HisRS chimeras, are composed of separate domains fused with a flexible linker [18,72]. Thus, such artificial enzymes were far less active and precise than natural enzymes [18,72]. On the other hand, the ribozymes required pre-activated amino acid substrates rather than free amino acid and ATP [73]. An apparent drawback of my allo-tRNA strategy is that the tip of the V-arm is distant from the CCA tail of tRNA. It may be helpful to use a long and rigid linker motif found in mitochondrial AlaRS species [74] or to use a long-D-arm binding protein domain to wrap around tRNA [37,38]. The use of T-box riboswitches as RNA scaffolds may be an alternative approach [73,75]. In summary, the novel allo-tRNA chassis, or orthogonal allo-tRNAs, would facilitate creation of synthetic tRNA-aminoacylation enzymes and ribozymes.

## 4. Materials and Methods

### 4.1. Cloning tRNA and Protein Genes

The nucleotide and amino-acid sequences are provided in the supplementary Excel file. All tRNA sequences (including the CCA tail) were cloned between the EcoRI and BglII sites in the pBAD-RSF5 plasmid [26] re-constructed in this study. The *cat* variant genes were developed from pACYC184 using Infusion (TAKARA Bio Inc., Shiga, Japan). The Ca. M. alvus TyrRS gene was cloned into pACYC184 by replacing the *cat* gene ORF. The HisRS ORFs were cloned immediately downstream of the *tet* gene of pACYC184-cat(193HisTAG). The *bla* variant gene was inserted downstream of the *tet* gene of pACYC184-cat(Ser146TAG) using Infusion. The other protein genes were cloned into the pGEX-6P-1 vector (a gift from Kazuhisa Nakayama, Addgene plasmid # 61838) and expressed under control of the *tac* promoter. Oligo DNAs and synthetic protein genes were purchased from Eurofin Genomics Co., Ltd. (Tokyo, Japan).

### 4.2. In Vivo Reporter Assays

*E. coli* DH10B and NEB 10-beta (NEB) cells were transformed with the indicated combinations of plasmids. Before spotting assays, transformed cells were pre-cultured in liquid LB media in the presence of 0.01% l-arabinose and 10 μM IPTG, if required, for a few hours [26]. In typical experiments, cell cultures were diluted to OD_600_ = 0.05 and then incubated for three hours. After spotting cell cultures (2 μL each) on LB agar plates, the plates were incubated at 37 °C overnight or for a longer time. For the induction of sfGFP genes, the final concentration of IPTG was 100 μM.

## Supplementary Materials

Supplementary Materials can be found at https://www.mdpi.com/1422-0067/21/20/7793/s1. Figure S1: Cloverleaf structures of the eight starting amber suppressor (9/3) allo-tRNA variants other than the S001 variant (Figure 1B), Figure S2: Inefficient sequestration of allo-tRNA^UTu1^ (or S012), Figure S3: Sequestration of S012 derivatives, Figure S4: Examining synthetic tRNA^His^ variants, Figure S5: A preliminary screening experiment of several cognate pairs of RNA aptamers and binding protein domains towards enhancing the histidylation activity, Figure S6: Cm resistance of *E. coli* DH10B (NEB 10-beta) cells expressing the *cat(His193TAG)* reporter gene and the indicated combinations of HisRS and tRNA variants, Table S1: Screening of candidate chassis tRNAs by using *E. coli* DH10B cells expressing the *cat(Ser146TAG)* reporter gene on growth media containing chloramphenicol (Cm) at a fixed concentration of 34 µg/ml.

## Abbreviations

aaRS	aminoacyl-tRNA synthetase
SerRS	seryl-tRNA synthetase
AlaRS	alanyl-tRNA synthetase
TyrRS	tyrosyl-tRNA synthetase
HisRS	histidyl-tRNA synthetase
SelA	selenocysteine synthase
PSTK	*O*-phosphoseryl-tRNA kinase
sfGFP	superfolder green fluorescent protein
IPTG	isopropyl β-d-1-thiogalactopyranoside
SECIS	selenocysteine insertion sequence
bp	base pair
cp	coat protein
SUMO	small ubiquitin-like modifier
WH	winged helix
SL	stem loop
ORF	open reading frame
NTD	*N*-terminal domain
CTD	*C*-terminal domain
ABD	anticodon binding domain
CD	catalytic domain
