# Supplementary material for: Rational Design of Aptamer-Tagged tRNAs
Source: Int J Mol Sci. 2020 Oct 21;21(20):7793. doi: 10.3390/ijms21207793 (PMC7590224; doi:10.3390/ijms21207793)
Supplement: Supplementary file 1 [file ijms-21-07793-s001.zip › MukaiiJMS_SI.pdf]

Rational design of aptamer-tagged tRNAs

Takahito Mukai

Department of Life Science, College of Science, Rikkyo University, 3-34-1 Nishi-Ikebukuro, Toshima-ku, Tokyo, 171-8501, Japan; takahito.mukai@rikkyo.ac.jp

Table S1. Screening of candidate chassis tRNAs by using *E. coli* DH10B cells expressing the *cat(Ser146TAG)* reporter gene on growth media containing chloramphenicol (Cm) at a fixed concentration of 34 μg/ml. Cm resistance (Cm^R) is indicated with "+", "±", or "–".

tRNA pair	Cm^R
S001/A001	+ / +
S002/A002	– / +
S003/A003	± / +
S004/A004	+ / +
S005/A005	– / +
S006/A006	+ / +
S007/A007	+ / +
S008/A008	+ / +
S009/A009	+ / +
S010/A010	+ / +
S011/A011	+ / +
S012/A012	+ / +
S013/A013	+ / +
S014/A014	+ / +
S015/A015	+ / +
S016/A016	+ / +
S017/A017	+ / +
S018/A018	+ / +
S019/A019	+ / +
S020/A020	+ / –
S021/A021	+ / –
S022/A022	– / –
S023/A023	– / –
S024/A024	– / –

tRNA pair	Cm^R
S025/A025	+ / –
S026/A026	+ / +
S027/A027	– / –
S028/A028	– / –
S029/A029	– / –
S030/A030	– / –
S031/A031	+ / –
S032/A032	+ / +
S033/A033	– / +
S034/A034	– / +
S035/A035	+ / +
S036/A036	+ / +
S037/A037	– / –
S038	+
S0385	+
S039/A039	– / –
S040/A040	+ / –
S041/A041	+ / +
S042/A042	+ / +
S043/A043	+ / +
S044/A044	+ / +
S045/A045	+ / +
S046/A046	+ / +
S047/A047	+ / +

tRNA pair	Cm^R
S048/A048	+ / +
S049/A049	+ / +
S050/A050	+ / +
S051/A051	+ / +
S052/A052	– / –
S053/A053	+ / –
S054/A054	+ / +
S055/A055	+ / –
S056/A056	– / –
S057/A057	– / –
S058/A058	– / –
S059/A059	– / –
S060/A060	+ / +
S061/A061	+ / +
S062/A062	+ / +
S063/A063	+ / +
S064/A064	+ / +
S065/A065	+ / +
S066/A066	+ / +
S067/A067	+ / +
S068/A068	+ / +
S069/A069	– / –
S070/A070	– / –
S071/A071	– / –

tRNA pair	Cm^R
S072/A072	– / +
S073/A073	– / +
S074/A074	+ / +
S075/A075	+ / +
S076/A076	+ / –
S077/A077	+ / +
S078/A078	+ / +
S079/A079	– / +
S080/A080	– / +
S081/A081	– / +
S082/A082	+ / +
S083/A083	+ / +
S084/A084	+ / +
S085/A085	+ / +
S086/A086	– / +
S087/A087	+ / +
S088/A088	+ / +
S089/A089	+ / +
S090/A090	+ / –
S091/A091	+ / –
S092/A092	+ / –
S093/A093	– / –
S094/A094	– / –

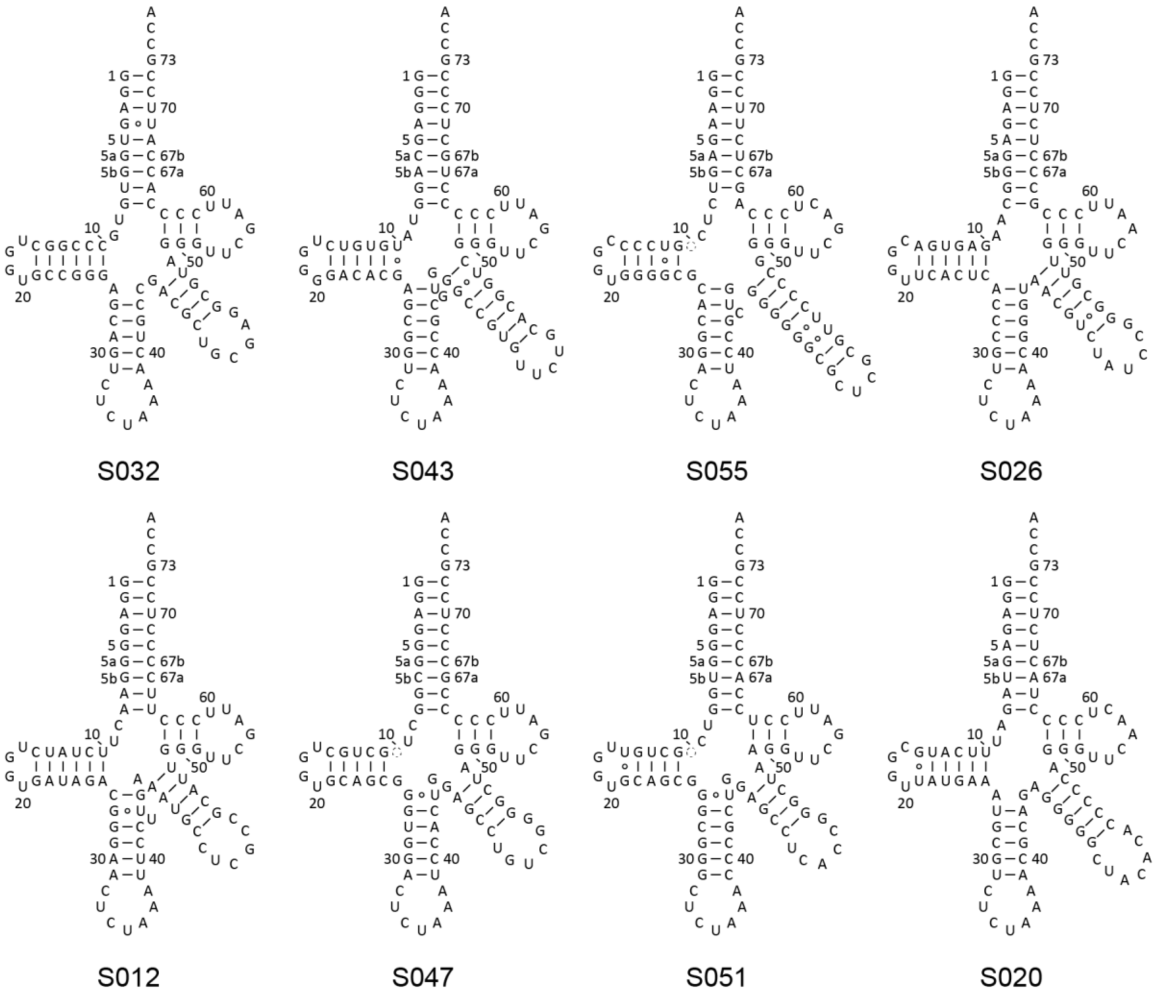

Figure S1. Cloverleaf structures of the eight starting amber suppressor (9/3) allo-tRNA variants other than the S001 variant (Figure 1B).

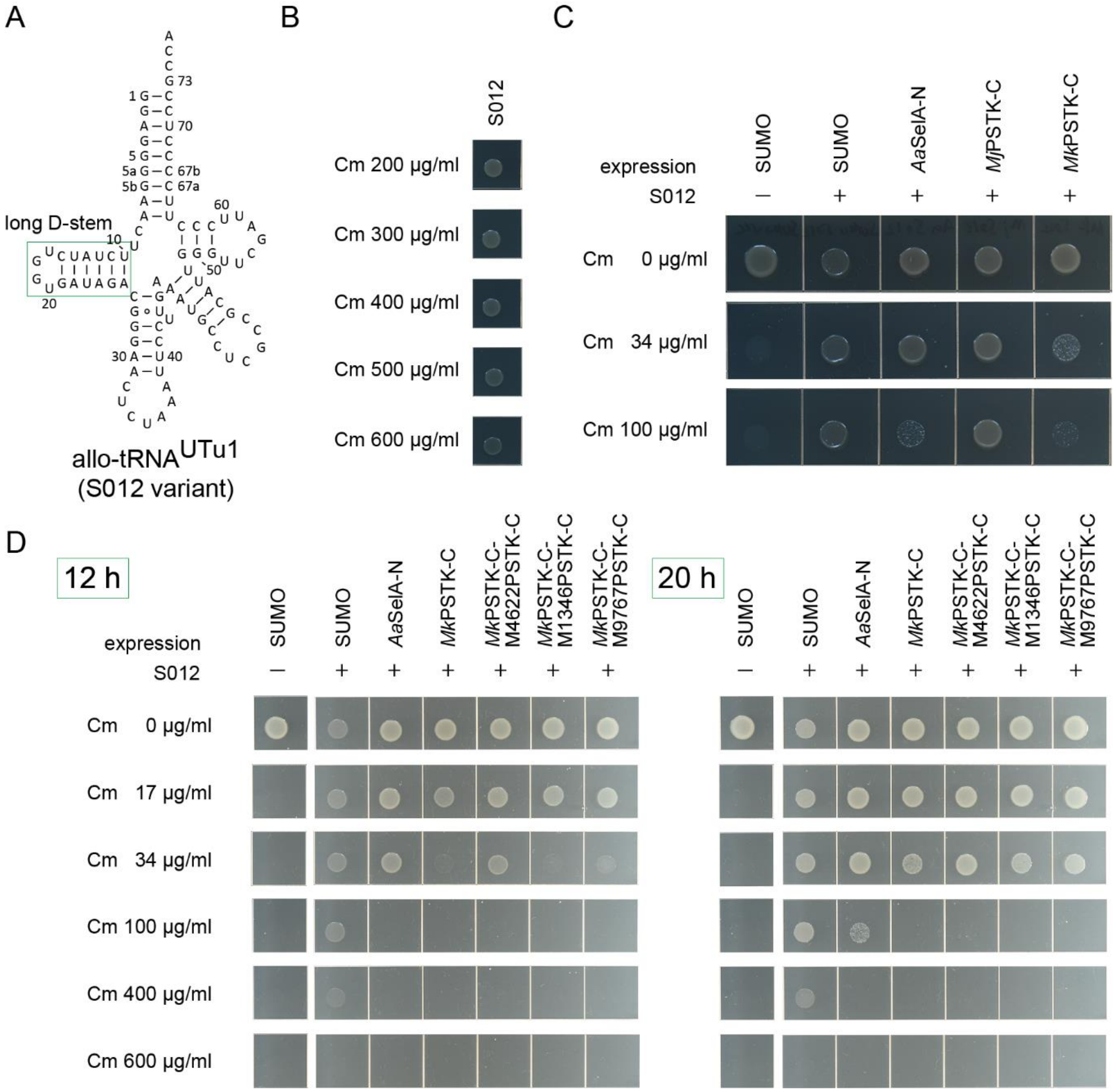

Figure S2. Inefficient sequestration of allo-tRNA^{UTu1} (or S012). (A) The cloverleaf structure of allo-tRNA^{UTu1} (or S012 in this study) which is as active tRNA^{Ser} as a native *E. coli* tRNA^{Ser} in *E. coli*. (B) S012 conferred almost maximum Cm resistance to *E. coli* cells expressing the *cat(Ser146TAG)* gene. (C) The S012 tRNA molecules were not efficiently sequestered by the N-terminal domain of *Aquifex aeolicus* SelA (*Aa*SelAN) or the C-terminal domain of *Methanopyrus kandleri* PSTK (*Mk*PSTK-C). (D) The S012 sequestration efficiency was not enhanced by expressing fusion proteins containing the *Mk*PSTK-C domain and another PSTK-C domain from three *Methanopyrus* species (M4622, M1346, and M9767). The *Mk*PSTK-C protein and the *Mk*PSTK-C-M1346PSTK-C fusion protein were the most effective inhibitor in this assay. The incubation times after cell spotting are shown.

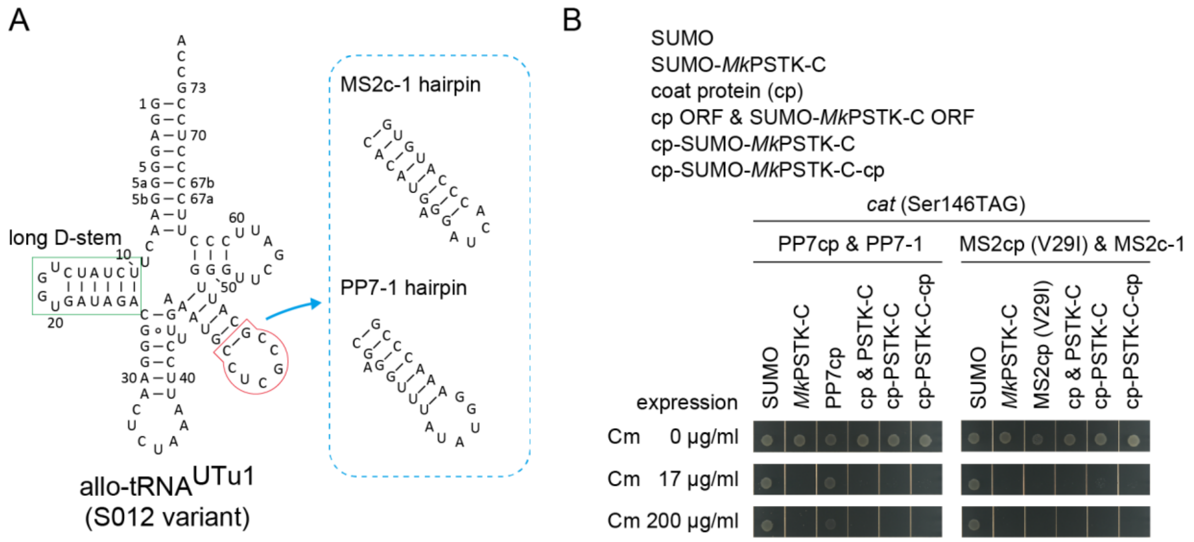

Figure S3. Sequestration of S012 derivatives. (A) The cloverleaf structure of S012 derivatives carrying either an MS2 C-loop hairpin or a PP7 hairpin. (B) The S012-PP7-1 variant was not efficiently sequestered by the PP7 coat protein, because the cells showed Cm 200 μg/ml resistance. In contrast, the S012-MS2c-1 variant was sequestered by the MS2 coat protein V29I variant. Information on the expressed proteins was shown above.

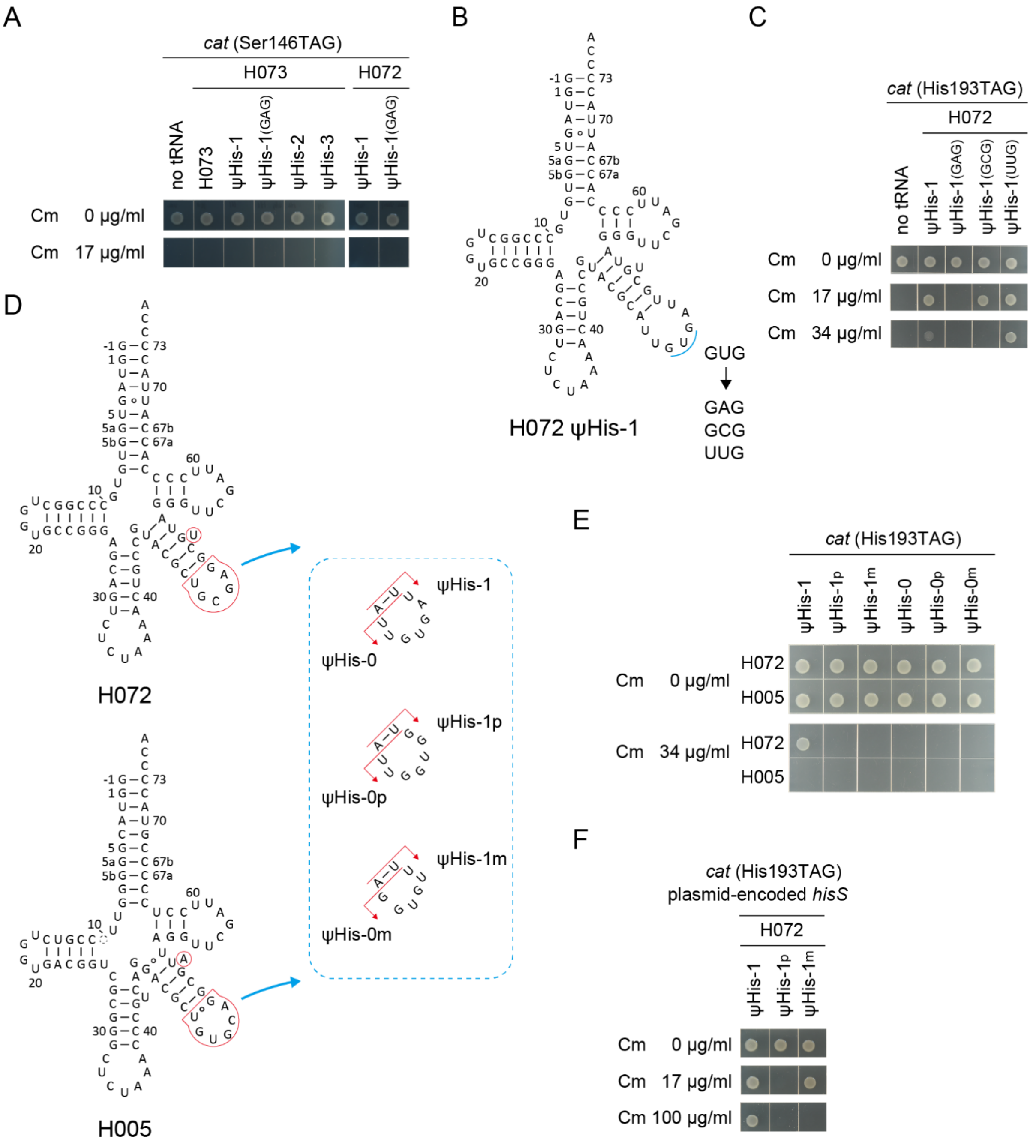

Figure S4. Examining synthetic tRNA^{His} variants. (A) The synthetic tRNA^{His} variants examined did not transfer Ser, since no Cm resistance was observed when the *cat(Ser146TAG)* reporter gene was used in *E. coli* DH10B cells. (B) The cloverleaf structure of H072 ψHis-1 variants. The GUG sequence in the V-arm loop was changed to GAG, GCG, or UUG. (C) Mutation in the V-arm loop affected the His-inserting activities of the H072 ψHis-1 variants. The original H072 ψHis-1 variant and its UUG variant conferred Cm 34 μg/ml resistance to *E. coli* DH10B cells expressing the *cat(His193TAG)* reporter gene. (D) The cloverleaf structures of the H072 and H005 variants and their derivatives. (E) Only the H072 ψHis-1 variant conferred Cm 34 μg/ml resistance to *E. coli* DH10B cells expressing the *cat(His193TAG)* gene. (F) A moderate overexpression of *E. coli* HisRS enhanced the His-inserting activity by the H072 ψHis1 variant. This is a part of Figure S5C.

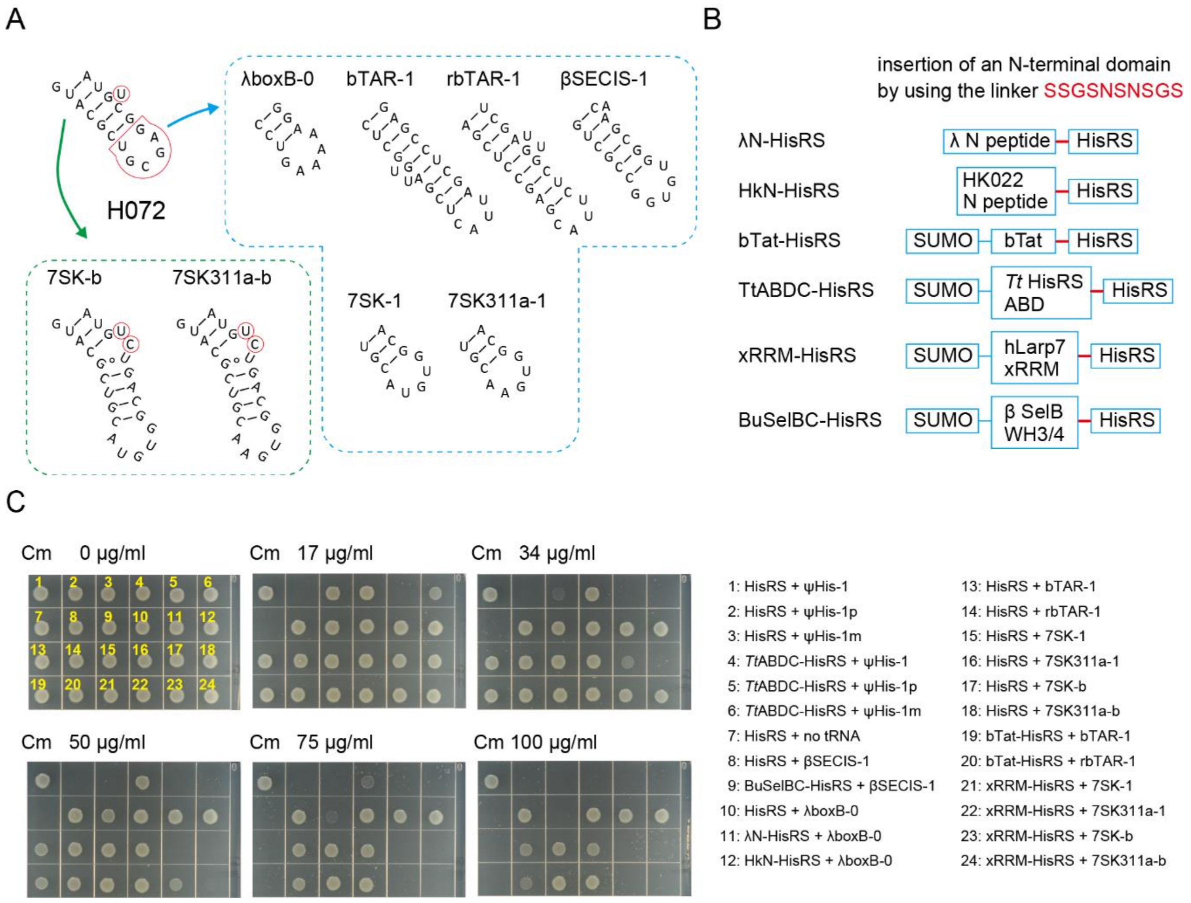

Figure S5. A preliminary screening experiment of several cognate pairs of RNA aptamers and binding protein domains towards enhancing the histidylation activity. (A) The secondary structures of transplanted aptamer RNAs. (B) The domain structures of *E. coli* HisRS variants fused with an N-terminal aptamer-binding protein domain. (C) Cm resistance of *E. coli* DH10B cells expressing the *cat(His193TAG)* reporter gene and the indicated combination of protein and tRNA.

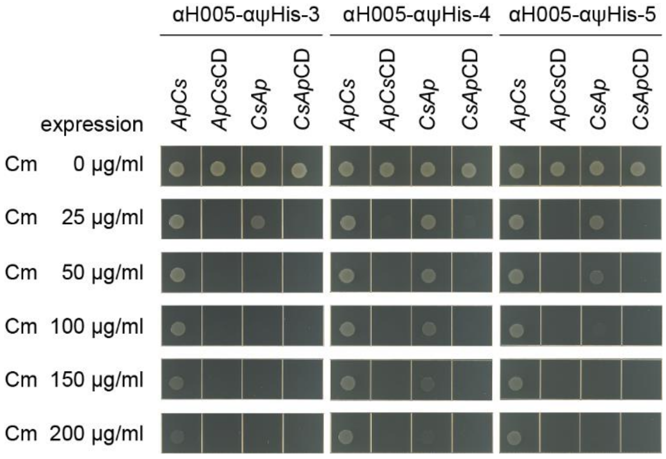

Figure S6. Cm resistance of *E. coli* DH10B (NEB 10-beta) cells expressing the *cat(His193TAG)* reporter gene and the indicated combinations of HisRS and tRNA variants.
